# Association of a high-fat diet with I-FABP as a biomarker of intestinal barrier dysfunction driven by metabolic changes in Wistar rats

**DOI:** 10.1186/s12944-023-01837-9

**Published:** 2023-05-27

**Authors:** Aisha Mahmood, Muhammad Naeem Faisal, Junaid Ali Khan, Humaira Muzaffar, Faqir Muhammad, Jazib Hussain, Jawad Aslam, Haseeb Anwar

**Affiliations:** 1 Department of Physiology, The Islamia University of Bahawalpur, Bahawalpur, 63100 Pakistan; 2 Institute of Physiology and Pharmacology, University of Agriculture, Faisalabad, 38040 Pakistan; 3 Faculty of Veterinary and Animal Sciences, Muhammad Nawaz Shareef University of Agriculture, Multan, Pakistan; 4 Department of Physiology, Government College University, Faisalabad, 38040 Pakistan; 5 Faculty of Veterinary Science, Bahaudin Zakariya University, Multan, Pakistan; 6 Department of Cellular and Molecular Medicine, University of Copenhagen, Copenhagen, Denmark

**Keywords:** High-fat diet, Metabolic disorders, Obesity, Dyslipidemia, Intestinal fatty acid binding protein, Insulin and leptin resistance

## Abstract

**Background:**

The epithelial lining of the gut expresses intestinal fatty-acid binding proteins (I-FABPs), which increase in circulation and in plasma concentration during intestinal damage. From the perspective of obesity, the consumption of a diet rich in fat causes a disruption in the integrity of the gut barrier and an increase in its permeability.

**Hypothesis:**

There is an association between the expression of I-FABP in the gut and various metabolic changes induced by a high-fat (HF) diet.

**Methods:**

Wistar albino rats (*n* = 90) were divided into three groups (*n* = 30 per group), viz. One control and two HF diet groups (15 and 30%, respectively) were maintained for 6 weeks. Blood samples were thus collected to evaluate the lipid profile, blood glucose level and other biochemical tests. Tissue sampling was conducted to perform fat staining and immunohistochemistry.

**Results:**

HF diet-fed rats developed adiposity, insulin resistance, leptin resistance, dyslipidemia, and increased expression of I-FABP in the small intestine compared to the control group. Increased I-FABP expression in the ileal region of the intestine is correlated significantly with higher fat contents in the diet, indicating that higher I-FABP expression occurs due to increased demand of enterocytes to transport lipids, leading to metabolic alterations.

**Conclusion:**

In summary, there is an association between the expression of I-FABP and HF diet-induced metabolic alterations, indicating that I-FABP can be used as a biomarker for intestinal barrier dysfunction.

**Supplementary Information:**

The online version contains supplementary material available at 10.1186/s12944-023-01837-9.

## Introduction

A well-balanced diet contains nutrients that are necessary for survival and maintenance of life. An association between changes in energy homeostasis and alterations in persistent food intake has been well elaborated earlier [1]. Dietary imbalances, such as those related to a high-fat (HF) diet, are linked to the epidemics of metabolic syndrome, hyperglycemia, obesity, hypertension, and dyslipidemia [2–4]. Obesity is pathologically evident as a result of an imbalance between the intake and expenditure of energy. Various dietary regimens, especially a HF diet, have been used as common tools for inducing obesity [5]. Dietary lipids are transported to the whole body via the blood after absorption through enterocytes. These lipids are, in fact, a diverse group of hydrophobic molecules, such as phospholipids, cholesterol, fatty acids (FAs), triglycerides (TGs), fat-soluble vitamins and ceramides [6]. Pancreatic enzymes are specifically responsible for the hydrolysis of these dietary lipids, such as the conversion of TGs into monoglycerides and FAs. In the small intestine, fat absorption takes place in the epithelial cells of enterocytes, whereas the transport of FAs takes place through cytosolic FABP [7], an intracellular protein expressed in various metabolically active organs (muscle, heart, brain, intestine and liver), which, in turn, has a vital role in the transportation and metabolism of long-chain FAs [8].

Fatty acid-binding proteins (FABPs) are expressed in high concentrations in different organs and are actively engaged in fat metabolism. To date, approximately nine coding genes for FABPs have been identified in humans, namely, heart (H-FABP), liver (L-FABP), intestine (I-FABP), epidermal (E-FABP), adipocyte (A-FABP), brain (B-FABP), myelin (M-FABP), ileal (Il-FABP), and testes (T-FABP). Among these, the I-FABP (I-FABP and FABP2) exist abundantly and specifically in the epithelium of the small intestine within villi but not in intestinal crypts [6]. These I-FABP proteins can be used as biomarkers for specific organ tissue damage because I-FABP is a soluble and stable molecule at room temperature, and its concentration in serum and urine increases as a result of intestinal damage, even at the early stage [9, 10]. The use of I-FABP as a biomarker of intestinal damage has been extensively studied using enzyme-linked immunosorbent assays (ELISAs) in cattle, humans, and pigs. However, the mechanism regarding the regulation of subcellular localization of FABPs and the potential actions of I-FABPs are poorly understood. The present study was therefore designed with the objective of ascertaining an association between the expression of I-FABP in the gut and various metabolic changes induced by a high-fat (HF) diet and assessing the appropriateness of I-FABP as a possible biomarker of intestinal inflammation and damage.

## Materials and methods

### Animals and diet

Six-week-old Wistar albino rats (*n* = 90) were used for the current study. Rats were acclimated for one week, during which rats were fed a normal basal diet, provided with ad libitum drinking water and kept on a light–dark cycle of 12:12 h. Post adaptation period, all rats were divided into three groups, with 30 rats in each group. Group one served as a control and received a normal basal diet only. The second and third groups were fed 15% and 30% fat, respectively, in addition to the normal basal diet (fat in the normal basal diet + 15% and 30% margarine). The composition of the normal basal diet and HF diets are given below.

## Composition of diets supplemented to study rats

**Table:** 

**Nutrients**	
Metabolizable Energy	3000 KCal/kg
Dry Matter	**%age** 88.8
Crude Protein	23
Crude Fiber	4.12
Crude Fat	4.19
Phosphorous	0.33
Calcium	0.90
Dig. Methionine	0.45
Dig. Arginine	1.09
Dig. Valine	0.79
Dig. Lysine	1.10
Dig. Tryptophan	0.20
Dig. Threonine	0.78
Dig. Isoleucine	0.79
**HF-diet for group 2**	Normal basal diet + 15% Margarine
**HF-diet for group 3**	Normal basal diet + 30% Margarine

At the end of the trial, the study rats were anesthetized with a combination of medetomidine and ketamine (1 and 50 mg/kg, respectively) maintained with isoflurane. Animals were sacrificed, and blood samples were obtained in serum-collection vacutainers. Serum was extracted through centrifugation and analyzed for various biochemical tests.

Most often IBD (inflammatory bowel disease) affects a portion of the small intestine just before the large intestine; i.e., ileum. It also affects the colorectal region of the large intestine. Therefore, tissue samples from these regions of the intestine (ileum and colorectal regions) were collected to perform fat staining and immunohistochemistry (*n* = 6 rats per group).

### Physical parameters

Weekly feed intake, defecation rate, and fecal pellet pH were monitored for each group using a pH meter (HANNA Instruments®; H12210). To measure the fecal defecation rate for each group, a daily recording of the fecal pellet number was performed, and then the weekly fecal defecation rate was calculated for each group from the collected data.

### Body weight and organ-to-body weight ratio

The body weight for all animals was recorded for each group during the entire experimental period. The body weight ratios of the pancreas, intestine, and abdominal fat were estimated using the formula given below:

$$\mathrm{Organ to body weight ratio}= \frac{\mathrm{Organ weight in grams}}{\mathrm{Body weight in grams}} \mathrm{X }100$$

### Biochemical analyses

Serum samples were analyzed to measure serum glucose, insulin, leptin, amylin, glucokinase, total cholesterol (TC), TGs, low-density lipoproteins (LDLs) and high-density lipoproteins (HDLs) through relative commercial kits as given below:

- Serum glucose: Bioclin® Glucose Monoreagent diagnostic kit, Berlin, Germany; detection limit range: 2-500 mg/dL; CV% < 3.1
- Serum Insulin: Calbiotech Insulin ELISA^®^, CA, USA; detection limit range: 0.78-50 µIU/mL; CV% < 10.
- Serum Leptin: Rat-LEP ELISA kit, E-EL-R0582, Thermo Fisher, Germany; detection limit range: 0.16~10 ng/mL; CV% < 10.
- Serum Amylin: Rat-Islet Amyloid Polypeptide ELISA kit, E-EL-R2448, Thermo Fisher, Germany; detection limit range: 62.50-4000 pg/m; CV% < 10.
- Serum glucokinase: Rat-GCK (glucokinase) ELISA kit, E-EL-R0426, Thermo Fisher, Germany; detection limit range: 0.63-40 ng/mL; CV% < 10.
- Serum Total Cholesterol: Dia-Sys Diagnostic Systems USA, detection limit range 3-750 mg/dL; CV% < 10.
- Serum Triglycerides: Dia-Sys Diagnostic Systems USA, detection limit range: 1000 mg/d; CV% < 10.
- High-density and low-density lipoproteins: Randox, Randox Laboratories LTD, UK: detection limit range 20 to 129 mg/d; CV% < 10. The serum low-density lipoprotein (LDL) concentration was measured by using the formula:
  $$\mathrm{LDL}(\mathrm{mg}/\mathrm{dl})=\mathrm{TC}-(\mathrm{TG}-\mathrm{HDL})5$$

### Fat staining

After slaughter as per the recommended protocol, tissue samples of the ileal and colorectal regions were collected, and fat staining (through Sudan black staining) was performed to stain the granules of fat that accumulated in the enterocytes. The acidic groups of compound lipids containing phospholipids combine with Sudan black satin because of the slightly basic nature of the dye.

### Immunohistochemistry

Immunohistochemistry was performed to determine the expression of I-FABP in the gut (ileum and colorectal regions). For this purpose, 5 μm sections of the intestine were fixed in formalin and embedded in paraffin, and then these intestinal sections were mounted on slides. The sections were deparaffinized, hydrated, and washed with phosphate buffer saline (PBS) at pH 7.4, and then, to block nonspecific binding sites, these sections were incubated with UltraCruz® Blocking Reagent (sc-516214) for blocking. Next, the primary antibody was incubated overnight with mouse anti-I-FABP antibody (sc-374482, Santa Cruz Biotechnology, USA) and then diluted with blocking reagent (1:200) at a temperature of 4 °C. Prepared slides were then washed and incubated after adding a secondary antibody, IgGκ BP-HRP anti-mouse antibody, sc-516102, Santa Cruz Biotechnology, Santa Cruz, USA, diluted with a blocking reagent (1:200) for a period of two hours. The visualization of the immunohistochemical reaction was performed by using the substrate, 3,3-di-amino-benzidine tetra-hydrochloride (DAB): sc-24982, Santa Cruz Biotechnology, and Immuno-Cruz® ABC kit, sc-516216. Dehydration was finally performed in a graded series of alcohol and xylene. The tissue sections were mounted with Ultra-Cruz™ mounting medium, sc-24941, Santa Cruz Biotechnology and cover-slipped.

### Statistical analysis

The obtained results were then subjected to a two-way analysis of variance (ANOVA), considering both the effect of days and HF diet treatment, followed by Duncan’s multiple range (DMR) test, and the results are shown as the mean ± SE. GraphPad Prism and Co-Stat software were used for statistical analysis.

## Results

Comparable results were observed between the different dietary fat groups of Wistar albino rats. The mean values for the control group were kept as a reference to compare the results of the other two groups.

### Physical parameters

**Fig. 1:**
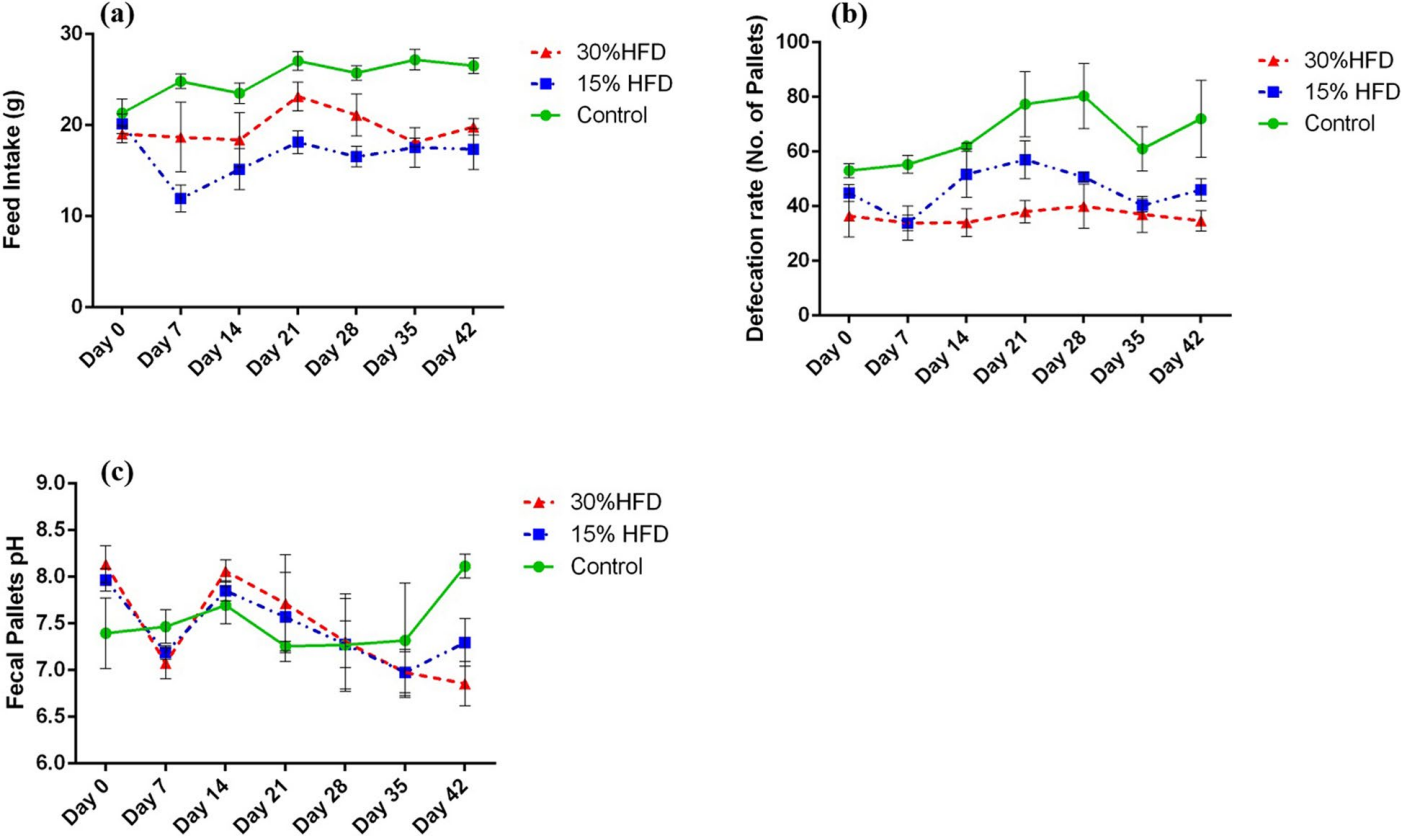
**a** Trendline for feed intake (mean ± SE, g), **b** defecation rate (mean ± SE), and **c** fecal pellet pH (mean ± SE) in the 15% and 30% HF diet-fed groups in comparison to the control group on different days

Feed intake (Fig. 1a) was significantly affected (*P* < 0.05) by the HF diet. The difference between the two HF-diet-fed groups was also statistically significant. The control group had the highest feed intake value, followed by the group fed a 15% HF diet and then the 30% HF diet-fed group. Statistical analysis demonstrated that the effect of the HF diet on the defecation rate (Fig. 1b) was also significant (*P* < 0.05). The difference between the two HF diet-fed groups was also statistically significant. The control group had the highest defecation rate, followed by the 15% and 30% HF diet-fed groups. A non-significant difference was observed with regard to fecal pellet pH between the three studied groups (Fig. 1c).

### Body weight and organ to body weight ratio

**Fig. 2:**
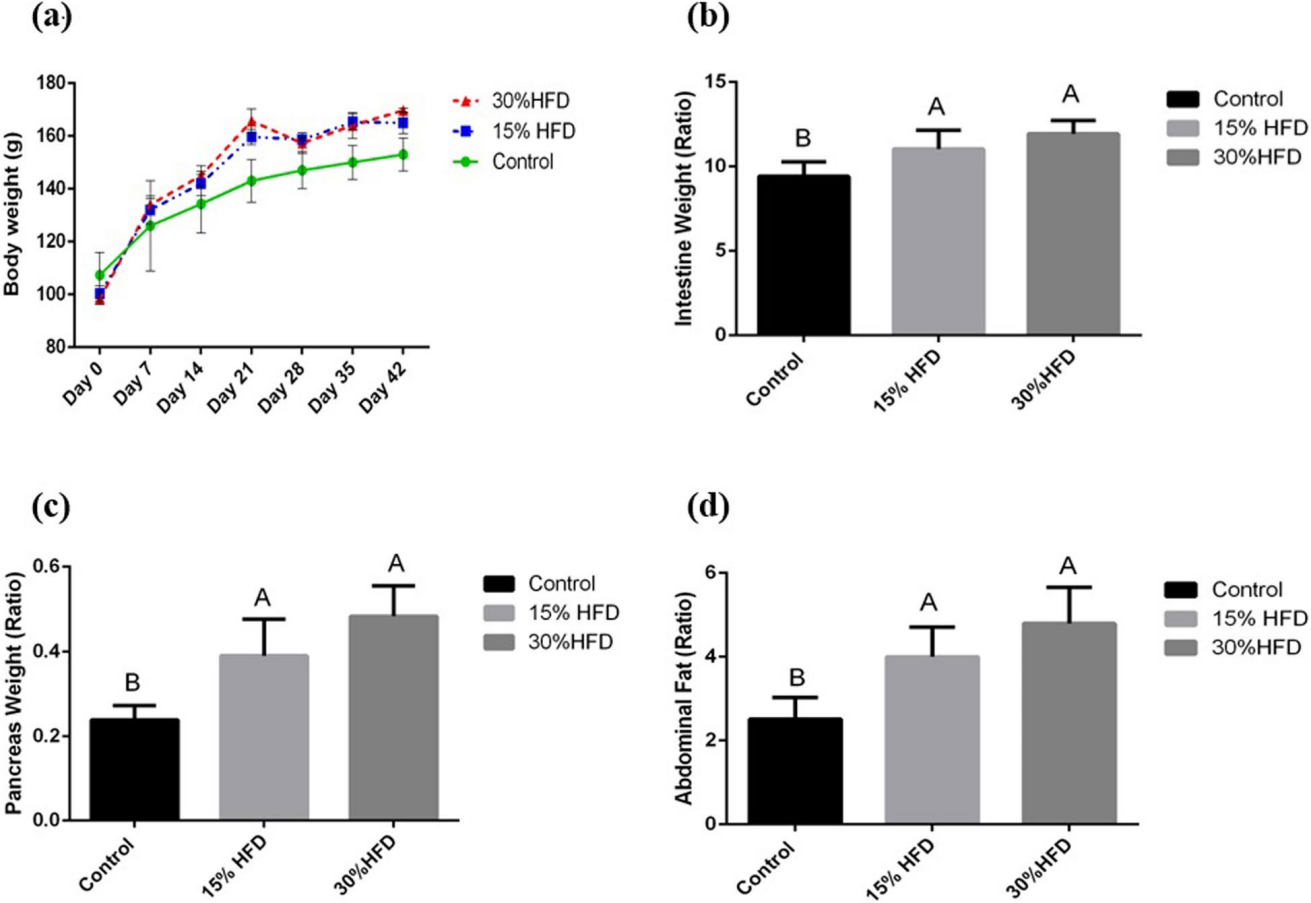
**a** Trendline for body weight (mean ± SE, g), **b** mean organ body weight ratio of the intestine, **c** pancreas, and **d** abdominal fat in the 15% HF-diet and 30% HF-diet-fed groups compared to the control group. ^**A−B**^ Means bearing different superscripts are significantly different from each other (*P* < 0.05)

After six weeks of the HF diet, weight gain was increased in the Wistar rats in both HF diet-fed groups (Fig. 2a). While weight gain was greater in the experimental groups fed 30% fat compared to the 15% HF-diet-fed group, this increase was non-significant. Rats in the 15% and 30% HF-diet groups exhibited a significantly higher (*P* < 0.05) intestine (Fig. 2b), pancreas (Fig. 2c), and abdominal fat (Fig. 2d) weight and body-weight ratio compared to the control group.

### Biochemical analyses

**Fig. 3:**
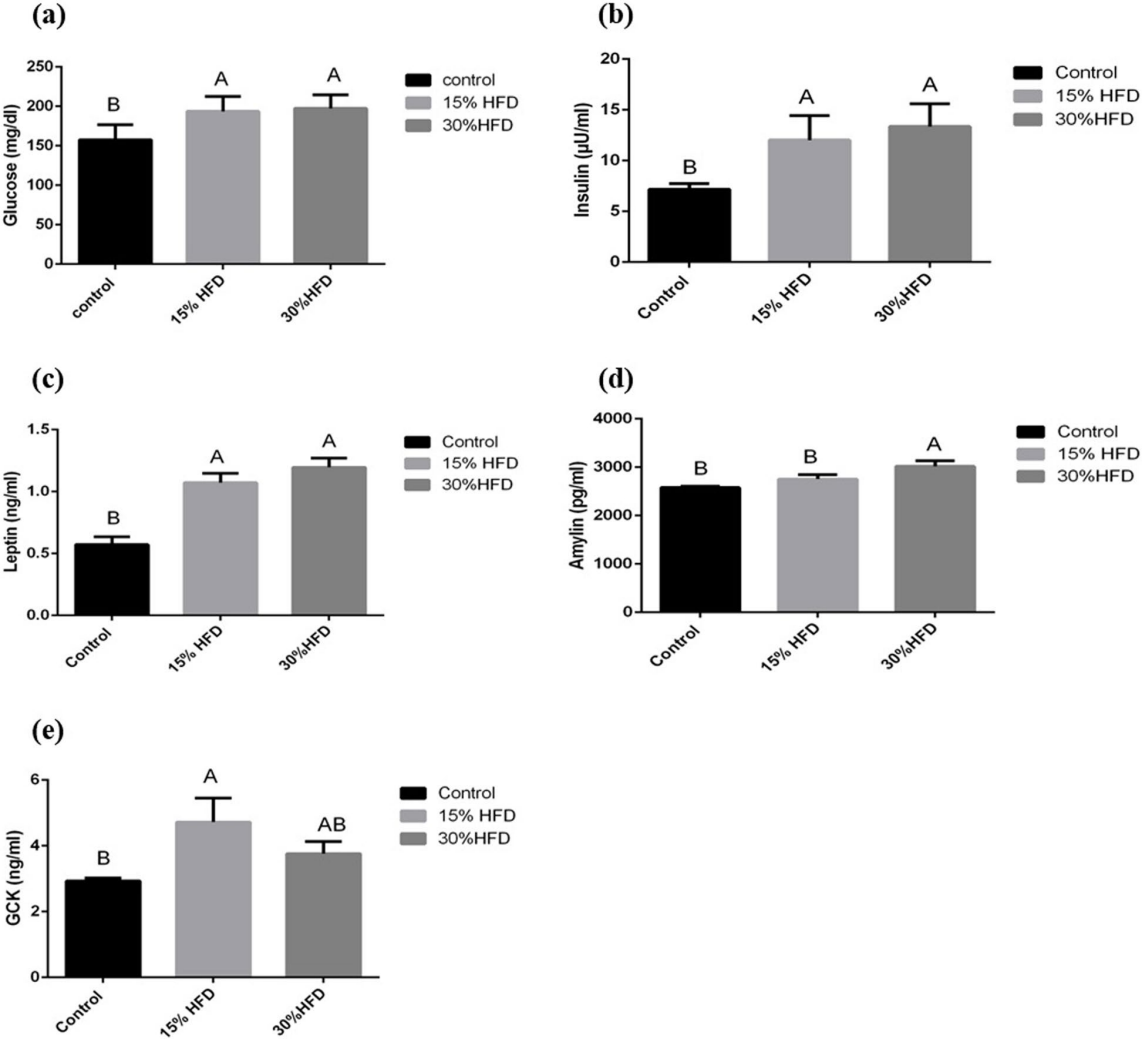
**a** Mean serum glucose concentration (mg/dl), **b** serum insulin levels (µIU/ml), **c** serum leptin levels (ng/ml), **d** serum amylin levels (pg/ml ± SE), and **e** serum GCK levels (mean ± SE, ng/ml) in the 15% and 30% HF diet-fed groups compared to the control group. ^**A−B**^ Means bearing different superscripts are significantly different from each other (*P* < 0.05)

Serum biochemical analyses showed that the mean concentrations of serum glucose (Fig. 3a), insulin (Fig. 3b), leptin (Fig. 3c), and GCK (Fig. 3e) were significantly higher (*P* < 0.05) in the HF diet-fed group than in the control group. The amylin level in serum (Fig. 3d) was significantly higher only in the 30% HF diet-fed group.

### Serum lipid profile

**Fig. 4:**
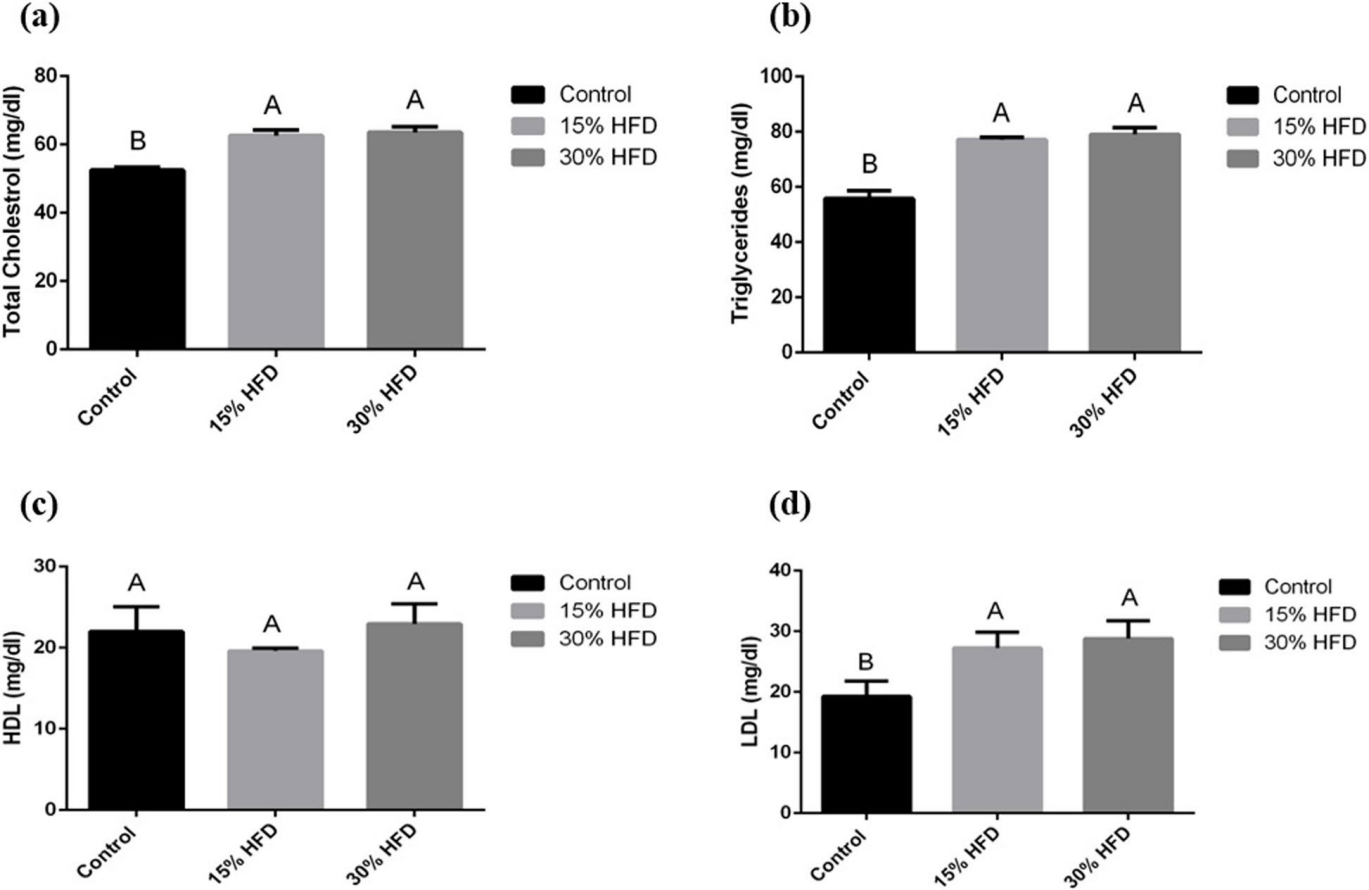
**a** Mean serum total cholesterol, **b** triglycerides, **c** HDL, and **d** LDL levels (mg/dl) in the 15% and 30% HF-diet-fed groups compared to the control group. ^**A−B**^ Means bearing different superscripts are significantly different from each other (*P* < 0.05)

The mean values of total cholesterol (Fig. 4a), TGs (Fig. 4b), and LDL-cholesterol (Fig. 4d) were significantly higher in the groups fed the HF-diet than in the control group. Serum HDL (Fig. 4c) was not significantly different among the studied groups.

### Fat staining

**Fig. 5:**
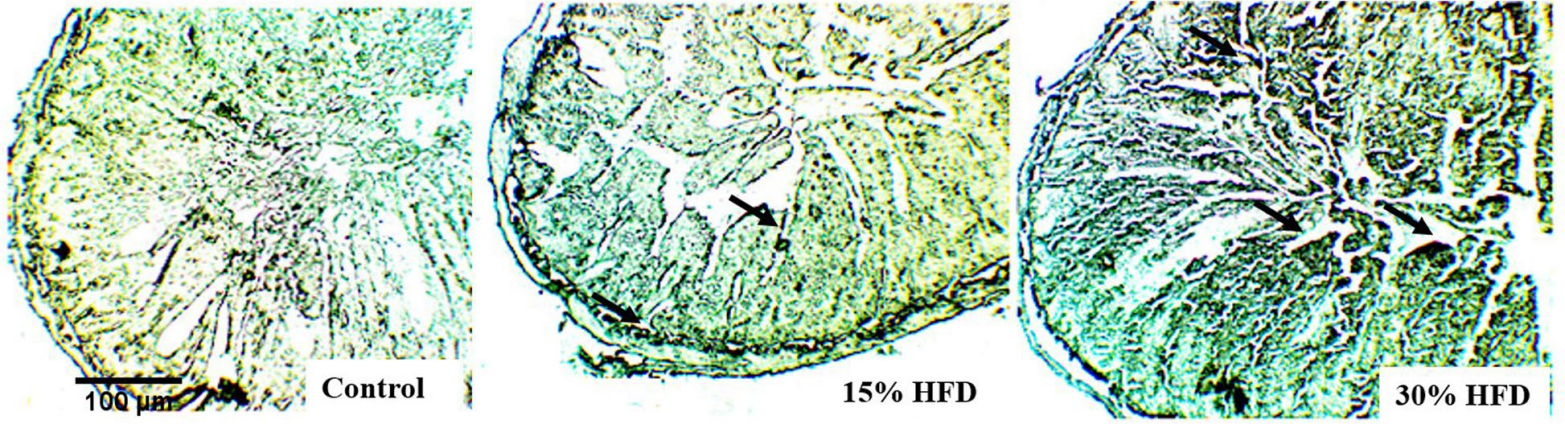
Photomicrographs of fat staining of the small intestine (ileum) of Wistar rats from the control group, 15% HF-diet group and 30% HF-diet-fed group (Sudan-Black staining; 10X); *n* = 6 rats per group

Photomicrographs of the intestinal tissue sections after fat staining (Sudan Black) are shown in Fig. 5. Animals from the 30% HF diet-fed group had more fat accumulation in their intestines than those fed a 15% HF diet (Fig. 5), indicating increased fat accumulation in a dose-dependent manner.

### Immunohistochemistry of I-FABP

**Fig. 6:**
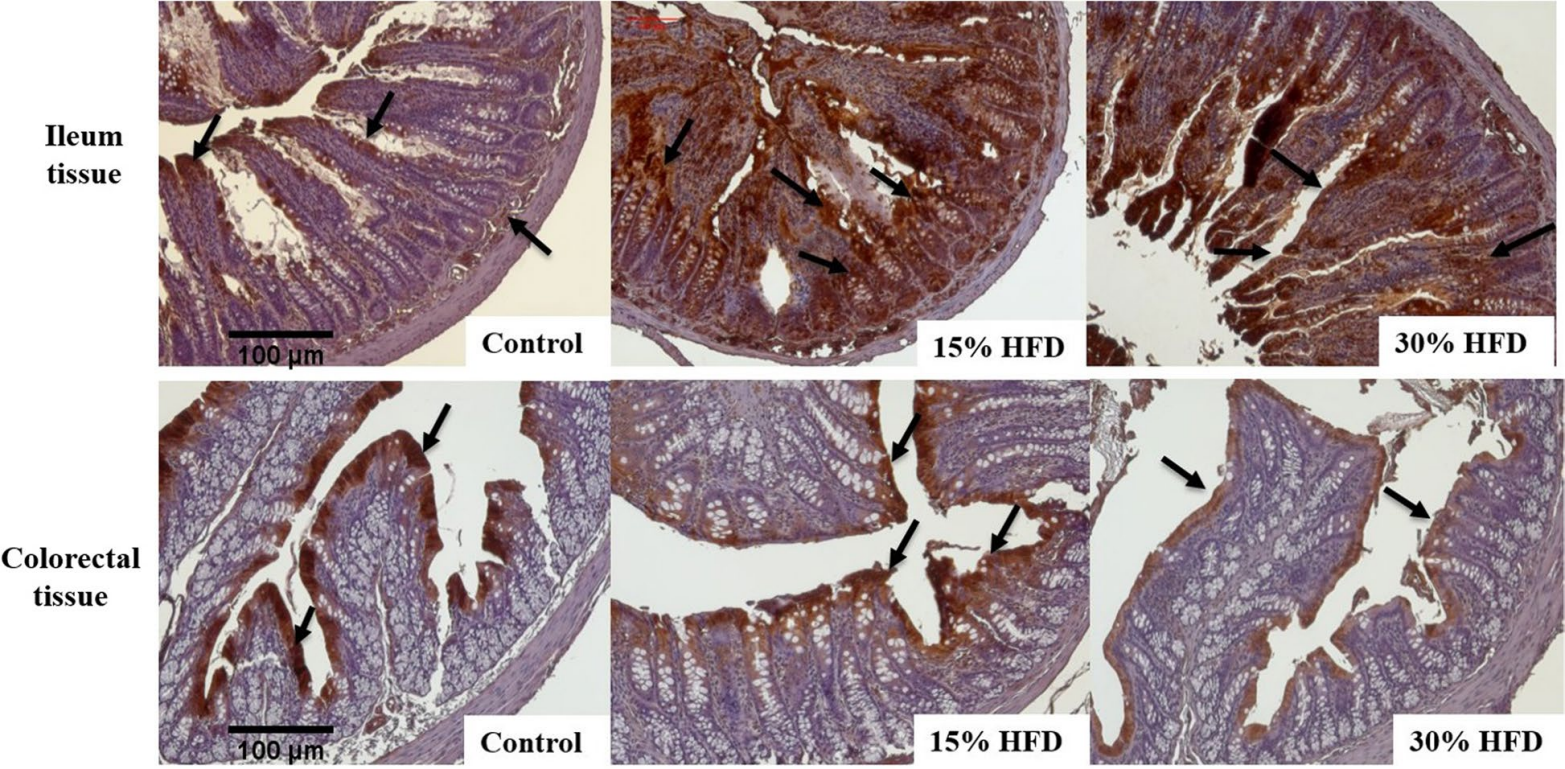
Photomicrographs indicating localization of I-FABP through immunohistochemistry in the small (ileum) and large intestine (colorectal area) of Wistar rats from the control group, 15% HF-diet group and 30% HF-diet-fed group (immunostaining; 10X). *n* = 6 rats per group

The results showed significant differences in I-FABP expression in the rat gut between the studied intestinal regions (Fig. 6). The localization of I-FABP was evident in the ileum in all layers of the intestinal wall, whereas expression of I-FABP in the colorectal region was observed only at the tip of the villus of the enterocytes. Moreover, higher I-FABP expression in the ileum of rats was present in the HF-diet-fed groups in comparison to the control group (Fig. 6). These alterations in I-FABP expression were more distinct in the ileum region of rats in all groups than in the colorectal region (Fig. 6). In the current study, increased expression of I-FABP in the ileum of high-fat diet-fed rats was correlated with increased fat intake, suggesting increased expression of I-FABP because of increased demand for lipid transport by enterocytes.

## Discussion

Intake of a HF diet is a crucial contributing factor in the development of metabolic disorders [11]. Various studies have provided evidence regarding the association of HF consumption with the development of metabolic changes, such as hyperinsulinemia, hypertriglyceridemia and hyperglycemia [12, 13]. Consistent with previous reports, the current study showed that HF diet-fed rats developed adiposity, elevated glucose, insulin resistance, leptin resistance, dyslipidemia and increased expression of I-FABP in the small intestine. Chronic elevations in serum glucose levels have been associated with increased loss of enterocytes, as assumed by elevated levels of I-FABP [14]. Increased loss of enterocytes in hyperglycemia is speculated to contribute to impaired gut integrity (disruption and destruction of the intestinal mucosal surface [4]), promoting inflammation [15].

I-FABP has emerged as a potential biomarker of gut barrier dysfunction in various gut-related diseases [16]. Any damage to gastrointestinal membranes may lead to a release of I-FABP into the blood, resulting in increased serum I-FABP concentration. Increased concentrations of plasma I-FABP indicate gut epithelial cell damage, while basal I-FABP levels might reflect the enterocyte physiological turnover rate [17]. However, data regarding gut barrier dysfunction and I-FABP in the case of metabolic diseases associated with a HF diet are limited. In this study, higher I-FABP expression in the ileum of HF-diet-fed rats was found, indicating increased I-FABP expression resulting from increased demand of enterocytes for lipid transport. This suggests that gut barrier dysfunction is associated with the ensuing metabolic changes.

The body weight and organ body weight ratio for intestine, pancreas and abdominal fat increased significantly in the high-fat diet-treated groups. These observations of the current study were supported by a past study summarizing that a high-fat diet significantly increases the accumulation of adipose tissue due to its high energy density, resulting in increased body weight and organ body weight ratio [18].

Elevated adipose mass (obesity) is associated with an increased concentration of leptin. Leptin is a hormone secreted by adipocytes and plays a very pronounced role in promoting energy expenditure and reducing appetite. A study [5] revealed leptin insensitivity development (high leptin levels fail to normalize body weight) [19] in rats after 8 weeks of HF diet feeding. Consistent with these results, it was also observed in the current study that significantly increased serum concentrations of leptin were associated with elevated fat mass in rats after consumption of a HF diet.

Previous studies have reported that dietary fat intake, both from plant (margarine) and animal origin, has resulted in considerable increases in serum total cholesterol (TC), LDL and TG. On the other hand, a decline in HDL concentration was found to be correlated with dietary butter and margarine intake [20], supporting the findings of the current study. Podrini et al. [21] also observed that HF diet intake resulted in increased plasma TC and LDL-cholesterol concentrations. Similar results have also been observed with a HF diet and increased serum TC, TG, free fatty acids (FFAs), and LDL levels [22].

The current study also revealed a significant effect on serum GCK levels, consistent with [22] and indicating that hepatic glucokinase is rapidly upregulated in response to HF diet intake (one week), contributing to the alteration in whole-body metabolism. Endogenous GCK upregulation caused by a HF diet tends to contribute to the development of obesity by modulating adaptive thermogenesis [23, 24]. An association between amylin and obesity has also been observed, suggesting high serum amylin levels due to HF intake. Another study [25] has also suggested that obesity can increase the secretion of hormones responsible for controlling food intake and body weight, such as pancreatic amylin and insulin (in obese humans and rodents).

### Statement of novelty

I-FABP has emerged as a potential biomarker of gut barrier dysfunction in numerous diseases related to the gut. However, this is the first time that the expression level of I-FABP was observed in the gut by tissue-based immunoassay using antibodies (immunohistochemistry) to assess the association of I-FABP expression in the gut with various metabolic changes induced by a high-fat diet.

### Strength of study

After a thorough literature review, it was found that this is the first report regarding the expression of I-FABP in the small and large intestine of rats, as shown through immunohistochemistry, to determine its association with metabolic changes as a result of a HF diet.

## Limitations of the study

The interaction of the expression of I-FABP with metabolic changes needs further study with respect to the molecular mechanisms involved.

## Conclusion

In conclusion, the results of this research demonstrate that Wistar rats show progressively elevated adiposity, hyperinsulinemia, hyperleptinemia, dyslipidemia, and increased expression of I-FABP in the gut (ileum) epithelium when challenged with a HF diet. These findings indicate that there exists a correlation between metabolic alterations and high expression of I-FABP in the intestine, suggesting that I-FABP could be useful as a diagnostic biomarker for intestinal barrier dysfunction. However, there is a need to conduct further research studies to reveal the molecular mechanisms of metabolic disease development in association with I-FABP levels and to discover other potential biomarkers for intestinal barrier dysfunction.

## Supplementary Information

**Additional file 1.**

## Data Availability

All datasets supplementary to the conclusions of this article have been incorporated in the article.

## References

[1] Friedman MI, Harris RB, Mattes RD (2008). Food intake: control, regulation and the illusion of dysregulation. appetite and food intake:. Behav Physiol Consid.

[2] Mao J, Hu X, Xiao Y, Yang C, Ding Y, Hou N, Wang J, Cheng H, Zhang X (2013). Overnutrition stimulates intestinal epithelium proliferation through β-catenin signaling in obese mice. Diabetes.

[3] Small L, Brandon AE, Turner N, Cooney GJ (2018). Modeling insulin resistance in rodents by alterations in diet: what have high-fat and high-calorie diets revealed?. Am J Physiol Endocrinol Metab.

[4] Mahmood A, Faisal MN, Khan JA, Muhammad F (2019). Margarine consumption induces oxidative stress in the gut of Wistar albino rats. Int J Agric Biol.

[5] Buettner R, Schölmerich J, Bollheimer LC (2007). High-fat diets: modeling the metabolic disorders of human obesity in rodents. Obesity.

[6] Gajda AM, Storch J (2015). Enterocyte fatty acid-binding proteins (FABPs): different functions of liver and intestinal FABPs in the intestine. Prostaglandins Leukot Essent Fatty Acids.

[7] Pang G, Xie J, Chen Q, Hu Z (2012). How functional foods play critical roles in human health. Food Sci Human Wellness.

[8] Cahyaningsih U, Satyaningtijas AS, Tarigan R, Nugraha AB. Chicken I-FABP as biomarker of chicken intestinal lesion caused by coccidiosis. InIOP Conference Series: Earth and Environmental Science 2018 Nov 1 (Vol. 196, No. 1, p. 012032). IOP Publishing.

[9] Glatz JF, Storch J (2001). Unraveling the significance of cellular fatty acid-binding proteins. Curr Opin Lipidol.

[10] Relja B, Szermutzky M, Henrich D, Maier M, De Haan JJ, Lubbers T, Buurman WA, Marzi I (2010). Intestinal-FABP and liver-FABP: Novel markers for severe abdominal injury. Acad Emerg Med.

[11] Lee CY (2013). The effect of high-fat diet-induced pathophysiological changes in the gut on obesity: what should be the ideal treatment?. Clin Transl Gastroenterol.

[12] Jiao Y, Wang X, Jiang X, Kong F, Wang S, Yan C (2017). Antidiabetic effects of Morus alba fruit polysaccharides on high-fat diet-and streptozotocin-induced type 2 diabetes in rats. J Ethnopharmacol.

[13] Petit V, Arnould L, Martin P, Monnot MC, Pineau T, Besnard P, Niot I (2007). Chronic high-fat diet affects intestinal fat absorption and postprandial triglyceride levels in the mouse. J Lipid Res.

[14] Verdam FJ, Greve JWM, Roosta S, van Eijk H, Bouvy N, Buurman WA, Rensen SS (2011). Small intestinal alterations in severely obese hyperglycemic subjects. J Clin Endocrinol Metab.

[15] Lau E, Marques C, Pestana D, Santoalha M, Carvalho D, Freitas P, Calhau C (2016). The role of I-FABP as a biomarker of intestinal barrier dysfunction driven by gut microbiota changes in obesity. Nutr Metab (Lond).

[16] Schurink M, Kooi EMW, Hulzebos CV, Kox RG, Groen H, Heineman E, Bos AF, Hulscher JBF. Intestinal fatty acid-binding protein as a diagnostic marker for complicated and uncomplicated necrotizing enterocolitis: a prospective cohort study. PLoS One. 2015;10(3):e0121336. 10.1371/journal.pone.0121336 PMC4368100 25793701

[17] Bischoff SC, Barbara G, Buurman W, Ockhuizen T, Schulzke JD, Serino M, Tilg H, Watson A, Wells J (2014). Intestinal permeability ¿ a new target for disease prevention and therapy. BMC Gastroenterol.

[18] Lim SM, Goh YM, Mohtarrudin N, Loh SP (2016). Germinated brown rice ameliorates obesity in high-fat diet induced obese rats. BMC Complement Altern Med.

[19] Pan WW, Myers MG (2018). Leptin and the maintenance of elevated body weight. Nat Rev Neurosci.

[20] Judd JT, Baer DJ, Clevidence BA, Kris-Etherton P, Muesing RA, Iwane M (2002). Dietary cis and trans monounsaturated and saturated FA and plasma lipids and lipoproteins in men. Lipids.

[21] Podrini C, Cambridge EL, Lelliott CJ, Carragher DM, Estabel J, Gerdin AK, Karp NA, Scudamore CL, Ramirez-Solis R, White JK (2013). High-fat feeding rapidly induces obesity and lipid derangements in C57BL/6 N mice. Mamm Genome.

[22] Wang C, Ha X, Li W, Xu P, Gu Y, Wang T, Wang Y, Xie J, Zhang J (2017). Correlation of TLR4 and KLF7 in inflammation induced by obesity. Inflammation.

[23] Tsukita S, Yamada T, Uno K, Takahashi K, Kaneko K, Ishigaki Y, Imai J, Hasegawa Y, Sawada S, Ishihara H, Oka Y (2012). Hepatic glucokinase modulates obesity predisposition by regulating BAT thermogenesis via neural signals. Cell Metab.

[24] Young A (2005). Inhibition of gastric emptying. Adv Pharmacol.

[25] Boyle CN, Rossier MM, Lutz TA (2011). Influence of high-fat feeding, diet-induced obesity, and hyperamylinemia on the sensitivity to acute amylin. Physiol Behav.

