# Supplementary material for: Association of a high-fat diet with I-FABP as a biomarker of intestinal barrier dysfunction driven by metabolic changes in Wistar rats
Source: Lipids Health Dis. 2023 May 27;22:68. doi: 10.1186/s12944-023-01837-9 (PMC10223920; doi:10.1186/s12944-023-01837-9)
Supplement: Supplementary file 1 — Additional file 1. [file 12944_2023_1837_MOESM1_ESM.pdf]

Association of I-FABP expression in the gut wit...

By: Haseeb Anwar

As of: Mar 27, 2023 8:05:04 PM

3,235 words - 35 matches - 22 sources

Similarity Index
9%

Mode: Similarity Report

paper text:

Association of I-FABP expression in the gut with various metabolic changes induced by high-fat diet in Wistar rats Abstract Background: The epithelial lining of the gut expresses

**the intestinal fatty-acid binding** proteins (**I**	22

-FABPs) which increase in circulation and in plasma concentration during an intestinal damage. From the perspective of obesity, the consumption of a diet rich in fat causes a disruption in the integrity of the gut barrier and an increase in its permeability. Hypothesis: There is an association between the expression of I-FABP in the gut with various metabolic changes induced by a high-fat (HF) diet. Methods: Wistar Albino rats (n=90) were

**divided into three groups (n** =30 **per group**) viz. one **control**	5

and two HF-diet groups (15 and 30%, respectively), maintained for 6 weeks. Results: It was revealed that HF-diet fed rats developed adiposity, insulin resistance, leptin resistance, dyslipidemia, and increased expression of I-FABP in the small intestine as

**compared to the control group** . Increased I-FABP **expression in the**	17

ileal region of the intestine is correlated significantly with higher fat contents in the diet, indicating that higher I-FABP expression occurs due to increased demand of enterocytes to transport lipids leading to metabolic alterations. Conclusion: In a nutshell, there is an association between the expression of I-FABP and the HF- diet-induced metabolic alterations, indicating that I-FABP can

**be used as a** biomarker for **intestinal barrier dysfunction**	2

. Key Words: High-Fat Diet, Metabolic Disorders, Obesity, Dyslipidemia,

Intestinal Fatty Acid Binding Protein , Insulin **and** 2

Leptin Resistance. Introduction A well-balanced diet contains nutrients which are necessary for survival and maintenance of life. An association between changes in energy homeostasis and alterations in persistent food intake has been well elaborated earlier [1]. Dietary imbalances, such as those related to a high-fat (HF) diet, are linked to the epidemics of metabolic syndrome; hyperglycemia, obesity, hypertension, and dyslipidemia [2-4]. Obesity is pathologically evident as a result of an imbalance between the intake and expenditure of energy. Various dietary regimens, especially a HF-diet, have been used as common tools for inducing obesity [5]. Dietary lipids are transported to the whole body via the blood after absorption through enterocytes. These lipids are, in fact, a diverse group of hydrophobic molecules, such as phospholipids, cholesterol, fatty acids (FAs), triglycerides (TGs), fat-soluble vitamins and ceramides [6]. The pancreatic enzymes are specifically responsible for the hydrolysis of these dietary lipids, such as the conversion of TGs, into monoglycerides and FAs. In the small intestine, the fat absorption takes place in the epithelial cells of enterocytes whereas, the transport of FAs takes place through cytosolic FABP [7]- an intracellular protein expressed in various metabolically active organs (muscle, heart, brain, intestine and liver), which, in turn, has a vital

role in the transportation **and** metabolism **of long-chain** FAs [8]. The **fatty** 19

-acid-binding proteins (FABPs) are expressed in high concentrations in different organs which are actively engaged in fat metabolism. Up till now, about nine coding genes for FABPs have been identified in humans namely heart (H-FABP),

liver (L-FABP), intestine (I-FABP), epidermal (E- **FABP), adipocyte (A-FABP**), brain (B- **FABP**), 6
myelin (M-FABP), **ileal (Il-FABP), and** testes (T- **FABP**). Among these, **the**

I-FABPs (I-FABP and FABP2) exist abundantly and specifically in the epithelium of small intestinal within their villi, but not in intestinal crypts [6]. These I-FABPs can be used as a biomarker for specific organ tissue damage, because I- FABP is a soluble and stable molecule at room temperature, and its concentration in serum and urine increases as a result of intestinal damage, even at the early stage [9, 10]. The use of I- FABPs as a biomarker of intestinal damage has extensively been studied using Enzyme Linked Immunosorbent Assay (ELISA) in cattle, humans, and pigs. However, the mechanism regarding the regulation of subcellular localization of FABPs and the potential actions of I-FABPs are poorly understood. The present study is therefore, being designed with the objective to ascertain an association between the expressions of I-FABP in the gut with various metabolic changes

induced by a high-fat (HF) **diet** and **to** 21

assess the appropriateness

of I-FABP as a possible biomarker **of intestinal** inflammation **and** damage. Materials **and** 2

methods Animals and Diet Wistar albino rats (n=90) of six-weeks of age were used for the current study. Rats were acclimated for one week, during which rats were fed a normal basal diet, provided with ad libitum drinking water and were kept on a light-dark cycle of 12:12 hours. Post

adaptation period , all **rats were divided into three groups** 1

, with 30 rats in each group. Group one served as a control and received a normal basal diet only. The second and third groups were fed with 15% and 30% of fat added in addition to the normal basal diet (fat in the normal basal diet + 15% and 30% margarine), respectively. The composition of the normal basal diet and HF-diets are given below: Composition of diets supplemented to study rats Nutrients Metabolizable Energy 3000 KCal/Kg %age Dry Matter. 88.8 Crude Protein Crude Fiber Crude Fat Phosphorous Calcium 23 4.12 4.19 0.33 0.90 Dig. Methionine Dig. Arginine Dig. Valine Dig. Lysine Dig. Tryptophan Dig. Threonine Dig. Isoleucine 0.45 1.09 0.79 1.10 0.20 0.78 0.79 HF-diet for group 2 HF-diet for group 3 Normal basal diet + 15% Margarine Normal basal diet + 30% Margarine

At the end of the trial, **the** study **rats were anesthetized** 16

with a combination of medetomidine and ketamine (1 and 50mg/kg, respectively) maintained with isoflurane. Animals were sacrificed and blood samples were attained in serum-collection vacutainers. Serum was extracted through centrifugation and analyzed for various biochemical tests. Most often IBD affects a portion of the small intestine just before the large intestine; i.e. ileum. It also affects the colorectal region of the large intestine. So, the tissue samples from these regions of the intestine (ileum and colorectal regions) were collected to perform fat staining and immunohistochemistry (n=6 rats per group). Physical parameters Weekly feed intake, defecation rate, and fecal pellet pH were monitored for each group. To measure the fecal defecation rate for each group, a daily recording of the fecal pellet number was done and then the weekly fecal defecation rate was calculated for each group from the collected data. Body Weight and organs to body weight ratio The body weight for all animals was recorded for each group during the entire experimental period. The body weight ratios of the pancreas, intestine, and abdominal fat were estimated using the formula given below: Organ to body weight ratio =

Organ weight in grams X 100 Body weight in grams Biochemical analyses Serum samples were analyzed for measuring serum glucose, insulin, leptin, amylin, glucokinase,

**total cholesterol (TC), TGs, low-density lipoproteins (LDLs**	) and	**high-density lipoproteins (HDLs**		11

) through relative commercial kits as given below: Serum Glucose: Bioclin® Glucose Monoreagent diagnostic kit, Berlin, Germany; detection limit range: 2-500 mg/dL; CV% < 3.1 Serum Insulin: Calbiotech Insulin ELISA®, CA, USA; detection limit range: 0.78-50 μIU/mL; CV% < 10. Serum Leptin: Rat-LEP ELISA kit, E-EL-R0582, Thermo Ficher, Germany; detection limit range: 0.16~10 ng/mL; CV% < 10. Serum Amylin: Rat-

**Islet Amyloid Polypeptide ELISA kit, E-EL-R2448**	8

, Thermo Ficher, Germany; detection limit range: 62.50-4000 pg/m; CV% < 10. Serum glucokinase:

**Rat-GCK (glucokinase) ELISA kit, E-EL-R0426**	20

, Thermo Ficher, Germany; detection limit range: 0.63-40 ng/mL; CV% < 10. Serum Total Cholesterol: Dia-Sys Diagnostic Systems USA, detection limit range 3 - 750 mg/dL; CV% < 10. Serum Triglycerides: Dia-Sys Diagnostic Systems USA, detection limit rang: 1000 mg/d; CV% < 10. High-density and low-density lipoproteins: Randox, Randox Laboratories LTD, UK: detection limit range 20 to 129 mg/d; CV% < 10. The serum low-density lipoproteins (LDLs) concentration was measured by using the formula: LDL (mg/dl) = TC – (TG – HDL) 5 Fat Staining After slaughter as per recommended protocol, tissue samples of the ileal and colorectal region were collected and fat staining (Through Sudan-black stain) was performed to stain the granules of fat which are accumulated in the enterocytes. The acidic groups of compound lipids containing phospholipids combine with Sudan black satin because of the slightly basic nature of the dye. Immunohistochemistry Immunohistochemistry was performed to find expression of I-FABPs both in the gut (ileum and colorectal regions). For this purpose, 5 μm sections of the intestine were fixed in formalin, embedded in paraffin and then these intestinal sections were mounted on slides. The sections were deparaffinized, hydrated, and washed with phosphate buffer saline (PBS) having

**pH 7.4**	, and then	**to block non-specific binding sites**	, these	**sections were incubated with**		9

UltraCruz® Blocking Reagent (sc-516214) for blocking. Following this, the primary antibody was incubated overnight with mice anti-I-FABPs antibody: sc-374482, Santa Cruz Biotechnology, USA and then diluted with blocking reagent (1:200) at a temperature of 4°C. Prepared slides were then washed and incubated after adding a secondary antibody:

IgGκ BP-HRP anti-mouse antibody, sc-516102, Santa Cruz Biotechnology 12

, Santa Cruz, USA, diluted with a blocking reagent (1:200) for a period of two hours. The visualization of the immunohistochemical reaction was done by using the substrate, 3, 3-di-amino-benzidine tetra-hydro-chloride (DAB): sc-24982, Santa Cruz Biotechnology, and Immuno-Cruz® ABC kit, sc-516216. Dehydration was finally done in a graded series of alcohol and xylene. The tissue sections

were mounted with Ultra-Cruz™ mounting medium, sc-24941, Santa Cruz Biotechnology 10

and cover-slipped.

Statistical analysis Obtained **results were** then **subjected to** a **two-way analysis of variance** 7 **(ANOVA**), considering both **the effect of** days **and**

HF-diet treatment,

followed by Duncan's multiple range (DMR) test and **results** 15

were shown as a mean ± SE. Graph-Pad prism and Co-Stat softwares were used for statistical analysis. Results Comparable results were observed between the different dietary fat groups of Wistar Albino rats. The mean value for the control group was kept as a reference to compare the results of the other two groups. Physical parameters Feed intake (Fig. 1a) was affected significantly ($P<0.05$) by HF-diet. The difference between the two HF-diet-fed groups was also statistically significant. The control group had the highest feed intake value, followed by the group fed with 15% HF-diet and then the 30% HF-diet-fed group respectively. Statistical analysis demonstrated that the effect of HF-diet on the defecation rate (Fig. 1b) was also significant ($P<0.05$). The difference between the two high-fat diet fed groups was also statistically significant. The control group had the highest defecation rate, followed by that in 15% and then 30% HF-diet-fed groups. A non-significant difference was observed with regard to fecal pellet pH between the three studied groups (Fig. 1c). Body Weight and organ to body weight ratio After six weeks of HF-diet, weight gain was increased in the Wistar rats in both HF-diet-fed groups (Fig. 2a). While weight gain was greater in the experimental groups fed 30% fat as compared to the 15% HF-diet-fed group, this increase was non-significant. Rats in the 15% and 30% HF-diet groups exhibited a significantly higher ($P < 0.05$) intestine (Fig. 2b), pancreas (Fig.2c), and abdominal fat (fig.2d) weight and body-weight ratio compared to the control group. Biochemical analyses Serum biochemical analyses showed that mean concentrations of serum glucose as shown in Fig. 3a,

insulin as shown in Fig. 3b, leptin (Fig. 3c), and GCK (Fig. 3e) were significantly higher (P < 0.05) in HF-diet fed group than that of the control group. Amylin level in serum (Fig. 3d) was significantly higher only in the 30% HF-diet-fed group. Serum lipid profile The mean values of total cholesterol (Fig. 4a), TGs (Fig. 4b), and LDL-cholesterol (Fig. 4d) were significantly higher in the groups fed with HF-diet than that in the control group. Serum HDL (Fig. 4c) was not different significantly amongst the studied groups. Fat Staining Photomicrographs of the intestinal tissue sections after fat staining (Sudan-Black) are shown in Figure 5. Animals from the 30% HF-diet-fed group had more fat accumulation in their intestines than those fed with a 15% HF-diet (Fig. 5), indicating increased fat accumulation in a dose- dependent manner. Immunohistochemistry of I-FABPs 8 Results showed significant differences in I-FABPs expression in the rat gut, between studied intestinal regions (Figure 6). The localization of I-FABPs was evident in the ileum in all layers of the intestinal wall, whereas, expression of I-FABPs in the colorectal region has been seen at the tip of the villus of the enterocytes only. Moreover, higher I-FABPs expression in the ileum of rats was present in HF-diet-fed groups in comparison to the control group (Fig. 6). These alterations in the I-FABPs expression were more distinct in the ileum region of rats, in all groups as compared to colorectal (Figure 6 B, B', B"). Discussion Intake of a HF-diet is a crucial contributing factor in the development of metabolic disorders [11]. Various studies have provided evidence regarding the association of HF consumption with the development of metabolic changes, such as hyperinsulinemia, hypertriglyceridemia, hyperglycemia, etc [12, 13,]. Consistent with previous reports, the current study presented that HF-diet-fed rats developed adiposity, elevated glucose, insulin resistance, leptin resistance, dyslipidemia and increased expression of I-FABP in the small intestine. Chronic elevations in serum glucose levels have been associated with increased loss of enterocytes, as assumed by elevated levels of I-FABP [14]. Increased loss of enterocytes in hyperglycemia is speculated to contribute to impaired gut integrity (disruption and destruction of the intestinal mucosal surface [4]), promoting inflammation [15].

I-FABP has emerged as a potential biomarker of gut **barrier dysfunction** 2

in various gut-related diseases [16]. Any damage to gastrointestinal membranes may lead to a release of I-FABP into the blood, resulting in increased serum I-FABP concentration. Increased concentrations of plasma I- FABP indicate gut epithelial cell damage, while

basal I-FABP levels might **reflect the** enterocyte 9 **physiological turnover rate** 2

[17]. However, data regarding gut barrier dysfunction and I-FABP in the case of metabolic diseases associated with HF-diet is limited. In this study, higher I-FABPs expression in the ileum of HF-diet-fed rats was found, indicating increased I-FABPs expression resulting from increased demand of enterocytes for lipid transport. This suggests that gut barrier dysfunction is associated with the ensuing metabolic changes. Elevated adipose mass (obesity) is associated with an increased concentration of leptin. Leptin is a hormone secreted by adipocytes and plays a very pronounced role in promoting energy

expenditure and reducing appetite. A study [5] has revealed leptin insensitivity development (high leptin level fails to normalize body weight) [18] in rats after 8 weeks of HF-diet feeding. Consistent with these results, it has also been observed

**in the current study that** significantly **increased** serum **concentrations of**	18

leptin were associated with an elevated fat mass in rats after consumption of the HF-diet. Previous studies have reported that dietary fat intake, both from plant (margarine) and animal origins has resulted in considerable increases in serum total cholesterol (TC), LDL and TG. On the other hand, a decline in HDL concentration was found to be correlated with dietary butter and margarine intake [19], supporting the findings of the current study. Podrini et al. [20] also observed that HF-diet intake resulted in increased plasma TC and LDL-cholesterol concentrations. Similar results have also been observed with a HF-diet and increased serum TC, TG, free fatty acids (FFAs), and LDL levels [21]. The current study also revealed a significant effect on serum GCK level, consistent with [21] and indicating that hepatic glucokinase is rapidly upregulated in response to a HF-diet intake (one week), contributing to the alteration in whole-body metabolism. Endogenous GCK upregulation caused by a HF-diet tends to contribute to developing obesity by modulating the adaptive thermogenesis [22, 23]. An association between amylin and obesity has also been observed, suggesting high serum amylin levels due to HF intake. Another study [24] has also suggested that obesity can increases the secretion of hormones responsible for controlling food intake and body weight such as pancreatic amylin and insulin (in obese humans and rodents). Statement of novelty I-FABPs have

**emerged as a potential biomarker of** gut **barrier dysfunction**	2

in numerous diseases related to the gut. However, this is the first time that the association of I-FABPs expression in the gut with various metabolic changes induced by HF-diet was studied. Strength of study After a thorough literature review, it was found that this is the first report regarding the expression of I-FABPs in the small and large intestines of rats, as shown through immunohistochemistry, to determine its association with metabolic changes as a result of HF-diet. Limitation of study The interaction of the expression of I-FABP with metabolic changes needs further study with respect to the molecular mechanisms involved. Conclusion In conclusion, the results of this research demonstrate that Wistar rats show progressively elevated adiposity, hyperinsulinemia, hyperleptinemia, dyslipidemia, and increased expression of I-FABP in the gut (ileum) epithelium when challenged with a HF-diet. These findings indicate that there exists a correlation between metabolic alterations and high expression of I-FABPs in the intestine, suggesting that I-FABPs could be useful as a diagnostic biomarker for intestinal barrier dysfunction. However, there is a need to conduct further research studies to reveal the molecular 11 mechanisms of metabolic disease development in association with I-FABPs and to discover other potential biomarkers for intestinal barrier dysfunction.

DECLARATIONS Ethical approval and consent to participate All procedures and protocols **were** 3

adopted under the guidelines permitted by the

institutional Bioethics Review **Committee, University of Agriculture, Faisalabad, Pakistan** 8

(Ref.# UAF/ORIC/7521)

for the use **and** care **of animals. Consent for publication Not Applicable. Availability of data materials** All **datasets** 3

supplementary for the conclusions of this article have been incorporated in the article.

Competing interests The authors claim to **have no competing interests. Funding** The **research was** financially supported **by the** Department **of** 4

Physiology, University of Agriculture, Faisalabad, Pakistan. Authors' contributions AM performed the major research work and data analysis. MNF was the major supervisor. JAK and FM supported the data analysis. HM, JH, and JA assisted in research and data analysis. HA provided technical support in the writing of the manuscript. Acknowledgements All of

the authors are highly obliged **to the University of Agriculture Faisalabad, Pakistan, for provision of technical support to perform this work** 1

.

The authors are also **thankful to the** Library 12 Department, **University of Agriculture Faisalabad** , and **the** 1

IT Department, Higher Education Commission (HEC, Islamabad) for the provision of access to books, journals and valuable databases. The authors thank Dr. Kaori Sakamoto for editing the manuscript and Penmanship company for

Grammar/syntax correction in the article. Figure Legends Fig. 1: (a) Trendline for feed intake (Mean ± SE, g), (b) defecation rate (Mean ± SE), and (c) fecal pellet pH (Mean ± SE) in the 15% and 30% HF-diet-fed

**groups in comparison to** the **control group** at different days. **Fig. 2** : (a) Trendline for **body weight**	1

(Mean ± SE, g), (b) mean organ body weight ratio of the intestine, (c) pancreas, and (d) abdominal fat in the 15% HF-diet

**and 30** % HF- **diet-fed groups compared to** the **control group**	1

. Fig. 3: (a) Mean

**serum glucose** concentration (**mg/dl), (b) serum insulin** levels (μIU/ **ml**), (c) **serum**	14

leptin levels (ng/ml), (d) serum amylin levels (pg/ml ± SE), and (e) serum GCK levels (Mean ± SE, ng/ml) in the

**15% and 30** % HF- **diet-fed groups compared to** the **control group**	1

. Fig. 4: (a) Mean serum

**total cholesterol, (b) triglycerides, (c) HDL, and (d) LDL** levels (mg/dl) **in** the 15% **and**	13

**30** % HF- **diet-fed groups compared to** the **control group**	1

. Fig. 5: Photomicrographs of fat staining of the small intestine (ileum) of Wistar rats from the control group, 15% HF-diet group and 30% HF-diet-fed group (Sudan-Black staining; 10X); n=6 rats per group Fig. 6: Photomicrographs indicating localization of I-FABPs through immunohistochemistry in the small (ileum) and large intestine (colorectal area) of Wistar rats from the control group, 15% HF-diet group and 30% HF-diet-fed group (immunostaining; 10X). n=6 rats per group 1 2 3 4 5 6 7 8 9 10 11 12 13 14 15 16 17 18 19 20 21 22 23 24 25 26 27 28 29 30 31 32 33 34 35 36 37 38 39 40 41 42 43 44 45 46 47 48 49 50 51 52 53 54 55 56 57 58 59 60 61 62 63 64 65 66 67 68 69 70 71 72 73 74 75 76 77 78 79 80 81 82 83 84 85 86

87 88 89 90 91 92 93 94 95 96 97 98 99 100 101 102 103 104 105 106 107 108 109 110 111 112 113 114 115 116 117 118 119 120 121 122 123 124 125 126 127 128 129 130 131 132 133 134 135 136 137 138 139 140 141 142 143 144 145 146 147 148 149 150 151 152 153 154 155 156 157 158 159 160 161 162 163 164 165 166 167 168 169 170 171 172 173 174 175 176 177 178 179 180 181 182 183 184 185 186 187 188 189 190 191 192 193 194 195 196 197 198 199 200 201 202 203 204 205 206 207 208 209 210 211 212 213 214 215 216 217 218 219 220 221 222 223 224 225 226 227 228 229 230 231 232 233 234 235 236 237 238 239 240 241 242 243 244 245 246 247 248 249 250 251 252 253 254 255 256 257 258 259 260 261 262 263 264 265 266 267 268 269 270 271 272 273 274 275 276 277 278 279 280 281 282 283 284 285 286 287 288 289 290 291 292 293 294 295 296 1 2 3 4 5 6 7 10 13

sources:

1 75 words / 2% - Internet from 08-Apr-2021 12:00AM
www.fspublishers.org

2 51 words / 1% - Crossref
Eva Lau, Cláudia Marques, Diogo Pestana, Mariana Santoalha, Davide Carvalho, Paula Freitas, Conceição Calhau. "The role of I-FABP as a biomarker of intestinal barrier dysfunction driven by gut microbiota changes in obesity", Nutrition & Metabolism, 2016

3 24 words / 1% - Internet from 11-Dec-2022 12:00AM
www.researchsquare.com

4 15 words / < 1% match - Internet from 30-Jan-2023 12:00AM
www.researchsquare.com

5 8 words / < 1% match - Internet from 09-Dec-2022 12:00AM
www.researchsquare.com

6 17 words / < 1% match - ProQuest
Yang, Diwen. "Identifying Endocrine-Disrupting Chemicals Binding to Human Nuclear Receptor and Intracellular Lipid-Binding Proteins", University of Toronto (Canada), 2022

7 16 words / < 1% match - Internet from 31-Jan-2023 12:00AM
www.frontiersin.org

8 16 words / < 1% match - Internet from 03-May-2021 12:00AM
www.pvj.com.pk

9 13 words / < 1% match - Internet
Front Neuroanat. 2014 Feb 12; 8:2

10 12 words / < 1% match - Crossref
M.E.R. Alebrante, M.M. Dias, C.A. Neves, D.D. Rosa, N.P. Siqueira, A.J. Natali, M.C.G. Peluzio. "Effects of exercise training and nebivolol treatment on atherosclerotic plaque development, iNOS expression and antioxidant capacity in apoE −/− mice", Science & Sports, 2018

11 12 words / < 1% match - Internet from 04-Feb-2023 12:00AM
www.mdpi.com

12 11 words / < 1% match - Crossref
Suleyman Arziman, Ozgur Tanriverdi, Seren Kucukvardar, Neslihan Citil, Aysegul Yildiz. "Salicylidene acylhydrazides attenuate survival of SH-SY5Y neuroblastoma cells through affecting mitotic regulator Speedy/RINGO and ERK/MAPK–PI3K/AKT signaling", Medical Oncology, 2020

13 11 words / < 1% match - Internet from 25-Sep-2022 12:00AM
unsworks.unsw.edu.au

14 9 words / < 1% match - Crossref
B. Dong, P. K. Saha, W. Huang, W. Chen et al. "Activation of nuclear receptor CAR ameliorates diabetes and fatty liver disease", Proceedings of the National Academy of Sciences, 2009

15 9 words / < 1% match - Internet from 15-Dec-2021 12:00AM
docsdrive.com

16 9 words / < 1% match - Internet from 22-Feb-2022 12:00AM
mdpi-res.com

17 8 words / < 1% match - Crossref
"Poster Sessions", Obesity Reviews, 05/2011

18 8 words / < 1% match - Crossref
Pascale Young, Isabella Russo, Paul Gill, Jane Muir, Rebekah Henry, Zoe Davidson, Ricardo J. S. Costa. "Reliability of pathophysiological markers reflective of exercise-induced gastrointestinal syndrome (EIGS) in response to 2-h high-intensity interval exercise: A comprehensive methodological efficacy exploration", Frontiers in Physiology, 2023

19 8 words / < 1% match - Crossref
Qigui Wang, Hui Li, Shuang Liu, Guihua Wang, Yuxiang Wang. "Cloning and Tissue Expression of Chicken Heart Fatty Acid-Binding Protein and Intestine Fatty Acid-Binding Protein Genes", Animal Biotechnology, 2005

20 8 words / < 1% match - Internet from 08-Mar-2023 12:00AM
u2.elabscience.com

21 7 words / < 1% match - Crossref
Agnieszka Owczarczyk-Saczonek, Joanna Czerwińska, Małgorzata Orylska, Waldemar Placek. "Effect of methotrexate treatment on the expression of epidermal-fatty acid-binding protein (E-FABP) and apolipoproteins in patients with psoriasis", Advances in Dermatology and Allergology, 2020

22

6 words / < 1% match - Crossref

Cellular Lipid Binding Proteins, 2002.
